# Ambroxol Hydrochloride Loaded Gastro-Retentive Nanosuspension Gels Potentiate Anticancer Activity in Lung Cancer (A549) Cells

**DOI:** 10.3390/gels7040243

**Published:** 2021-11-30

**Authors:** Shadab Md, Samaa T. Abdullah, Nabil A. Alhakamy, Ahmad Bani-Jaber, Ammu Kutty Radhakrishnan, Shahid Karim, Naiyer Shahzad, Gamal A. Gabr, Abdulmohsin J. Alamoudi, Waleed Y. Rizg

**Affiliations:** 1 Department of Pharmaceutics, Faculty of Pharmacy, King Abdulaziz University, Jeddah 21589, Saudi Arabia; nalhakamy@kau.edu.sa (N.A.A.); wrizq@kau.edu.sa (W.Y.R.); 2 Center of Excellence for Drug Research & Pharmaceutical Industries, King Abdulaziz University, Jeddah 21589, Saudi Arabia; ajmalamoudi@kau.edu.sa; 3 Mohamed Saeed Tamer Chair for Pharmaceutical Industries, King Abdulaziz University, Jeddah 21589, Saudi Arabia; 4 Department of Biological Sciences, Faculty of Science, King Abdulaziz University, Jeddah 21589, Saudi Arabia; 5 Department of Pharmaceutics and Pharmaceutical Technology, School of Pharmacy, The University of Jordan, Amman 11942, Jordan; Abjaber@ju.edu.jo; 6 Jeffrey Cheah School of Medicine and Health Sciences, Monash University, Subang Jaya 47500, Malaysia; ammu.radhakrishnan@monash.edu; 7 Department of Pharmacology, Faculty of Medicine, King Abdulaziz University, Jeddah 21589, Saudi Arabia; skaled@kau.edu.sa; 8 Department of Pharmacology and Toxicology, Faculty of Medicine, Umm Al-Qura University, Makkah 24382, Saudi Arabia; shahzad.naiyer@gmail.com; 9 Department of Pharmacology and Toxicology, College of Pharmacy, Prince Satam Bin Abdulaziz University, Al-Kharj 16278, Saudi Arabia; g.gabr@psau.edu.sa; 10 Department of Pharmacology and Toxicology, Faculty of Pharmacy, King Abdulaziz University, Jeddah 21589, Saudi Arabia

**Keywords:** alginate, ambroxol, kappa-carrageenan, nanosuspension, floating gels, sustained release, lung cancer

## Abstract

This study aimed to develop gastro-retentive sustained-release ambroxol (ABX) nanosuspensions utilizing ambroxol-kappa-carrageenan (ABX-CRG_K_) complexation formulations. The complex was characterized by differential scanning calorimetry, powder x-ray diffractometer, and scanning electron microscopy. The prepared co-precipitate complex was used for the development of the sustained-release formulation to overcome the high metabolic and poor solubility problems associated with ABX. Furthermore, the co-precipitate complex was formulated as a suspension in an aqueous floating gel-forming vehicle of sodium alginate with chitosan, which might be beneficial for targeting the stomach as a good absorption site for ABX. The suspension exhibited rapid floating gel behaviour for more than 8 h, thus confirming the gastro-retentive effects. Particle size analysis revealed that the optimum nanosuspension (ABX-NS) had a mean particle size of 332.3 nm. Afterward, the ABX released by the nanoparticles would be distributed to the pulmonary tissue as previously described. Based on extensive pulmonary distribution, the developed nanosuspension-released ABX nanoparticles showed significant cytotoxic enhancement compared to free ABX in A549 lung cancer cells. However, a significant loss of mitochondrial membrane potential (MMP) also occurred. The level of caspase-3 was the highest in the ABX-NS-released particle-treated samples, with a value of 416.6 ± 9.11 pg/mL. Meanwhile, the levels of nuclear factor kappa beta, interleukins 6 and 1 beta, and tumour necrosis alpha (NF-kB, IL-6, IL-1β, and TNF-α, respectively) were lower for ABX-NS compared to free ABX (*p* < 0.05). In caspase-3, *Bax*, and *p53*, levels significantly increased in the presence of ABX-NS compared to free ABX. Overall, ABX-NS produced an enhancement of the anticancer effects of ABX on the A549 cells, and the developed sustained-release gel was successful in providing a gastro-retentive effect.

## 1. Introduction

Lung cancer (LC) is a global health concern, and its occurrence rate continues to rise. Among the LCs, non-small cell lung cancer (NSCLC) is more common (85%) than small cell lung cancer (SCLC) (15%) [1]. Cigarette smoking is a significant risk factor for LC. Meanwhile, domestic biomass fuels, chronic obstructive pulmonary diseases, occupational exposures, air and environmental pollution, diet and nutrition, and genetic factors are also associated with LC [2]. A major concern with the LC is its very low survival rate. Treatment strategies used to treat LC include surgery, adjuvant therapy, chemotherapy, and radiotherapy; chemotherapy and radiotherapy are the common treatment options for NSCLC. It is recommended that a combination of cisplatin (or carboplatin) and a cytotoxic agent such as paclitaxel is used in chemotherapy [3]. Meanwhile, targeting signalling pathways have been explored against LC, and it appears to be promising [4]. Interestingly, synergistic effects between ambroxol (ABX) and chemotherapy agents such as paclitaxel and carboplatin have been reported in lung cancer [5]. ABX was shown to promote autophagosome accumulation by blocking late-stage autophagic flux in lung carcinoma cells. Inhibition of autophagy can potentiate anti-proliferative effects and contribute to tumour regression in lung carcinoma cells [6]. In addition, ABX provided peri-operative protection in lung cancer surgery [7]. Lung-protective effects and low toxicity are some of the major advantages of ABX use in LC therapy [6]. Moreover, ABX was approved for its antioxidant and anti-inflammatory actions when it was studied for treatment of hyperinflammatory diseases, such as psoriasis, and it was also suggested for treating cancer-associated hyperinflammatory status [8]. Furthermore, intravenous and oral ABX formulations accumulate in the lungs [9,10].

The high rate of ABX elimination was cited as the reason for its sustained release formulation to avoid repetitive dosing [11]. Meanwhile, avoidance of systemic side-effects, such as headache and insomnia, is the rationale used to support transdermal ABX delivery [12]. ABX has poor solubility, and oral bioavailability of around 80%, making it a good candidate for oral sustained delivery [9,10,11].

While ABX is a widely used expectorant, it has several other pharmacological actions, including anti-inflammatory and anti-viral effects. ABX is commercially available as tablets, capsules, dry powders, inhalation solutions, and liquid orals. Additionally, several advanced formulations of ABX have been studied and reported. For instance, a soft gel immediate-release formulation was developed for patients who have difficulty swallowing [13].

Meanwhile, plans to develop sustained-release formulations in the form of tablets and liquid orals are ongoing. Osmotic pump-based tablets of ABX with a comparable bioavailability as those found in marketed sustained-release capsule formulations can sustain drug release for 12 h [11]. Sustained ABX release can be obtained from microspheres prepared from the mucilage of *Abelmoschus esculentus* [14]. Cross-linking of microspheres can be utilized for modifying the release of ABX [15].

Interestingly, in situ oral gels of ABX using pectin solution provided sustained ABX release in the acidic medium of the stomach [16]. Bani-Jaber and Abdullah (2020) attempted to produce a sustained-release oral suspension of ABX through the complexation of a polymer and raft formation [17]. In addition, hydroxypropyl methylcellulose and poloxamer 407-based hydrogels are proving to be useful for transdermal ABX delivery [12].

The gastroretentive delivery system of ABX can overcome the problems associated with its low water solubility and high metabolism after oral administration [18]. The prolonged retention in the acidic pH of the stomach can enhance ABX solubility due to the positive charge that develops on the secondary amine of ABX [18]. Additionally, this pH enhances the absorption of ABX from the stomach by increasing the gastric residence time in the stomach, which is a good absorption site for ABX [18]. Meanwhile, several polymers, such as kappa-carrageenan (CRG_K_), sodium alginate (SA), and chitosan (CS) have been proven to be useful applications in the development of sustained release and gastroretentive delivery systems [19,20]. Carrageenans provide a robust controlled release system, but have problems of inadequate floating [20]. Fortunately, the use of a polymer blend containing CRG_K_, CS, and/or SA can overcome this obstacle. Moreover, SA and CS are also appropriate for this purpose to achieve sufficient floating. In addition, the inclusion of CS in the polymer blend has the specific advantages of immunological responses and mucoadhesive properties against cancer cells and can be presumed to complement the action of ABX against LC. In addition, the chitosan (CS) could enhance the general cell-mediated and humoral immunity after intestinal absorption when it became soluble at pH higher than 5. Afterward, CS could be considered as a biological modifier in the case of cancer due to the enhancement of the body immune cells (T-cells and natural killer cells) and antibody production to capture the cancer cells or the apoptotic cells after the absorbed ABX nanoparticles action [21]. The inclusion and release of the ABX were as nanoparticles from the suspension after raft/floating gel formation at the stomach. ABX nanoparticles will be absorbed at the stomach as proved to be distributed, especially at the lungs as one of ABX high distribution areas. From this fact, the released ABX nanoparticles were tested against the lung cancer cells. Additionally, the nanoparticles were found to have higher cancer cells internalization, better cancer cells sensitivity, and higher absorption at the gastric cells, as supported by the literature [5,6,7,8,9,10].

The present study aimed to develop sustained release nanosuspension floating gels of ABX by using co-precipitated complexes of ABX-CRG_K_, CS, and SA mixtures. The ABX nanosuspension (ABX-NS) was developed to form a coherent raft gel for up to 8 h during contact with simulated gastric fluid. The incorporation of SA and CS caused enhancement of the sustained release, ABX absorption, and ABX nanosuspension formation. The sustained release of ABX from the nanosuspension during the gastric residence time caused an increase in ABX absorption in the blood, which could further improve its bioavailability. Proof exists that oral ABX macro-suspensions have promising outcomes [17]. However, nanosuspensions as opposed to macro-suspensions are far better in solubility and bioavailability enhancement. Therefore, achieving additional benefits of formulation of nanosuspension was proposed in the study. While the kneading method is sufficient for macro-suspensions, the most suitable method to prepare nanosuspensions is co-precipitation using polymers [22]. Lambda-carrageenan has poor gelling and needs a higher concentration than kappa-carrageenan [23]. Thus, despite the reported use of lambda-carrageenan, kappa-carrageenan was chosen for the present study. The resulting SA gel strength was enhanced by incorporating CS instead of the excess lambda-carrageenan polymer. Thus, a sustained release fast floating gastroretentive gel-forming nanosuspension of ABX was prepared using co-precipitate complexation. In addition, the prepared ABX-NS were evaluated to enhance the anticancer activity in lung cancer cells compared to the free ABX (positive control), blank suspension without ABX, and the untreated cells (negative control) using the cell viability tetrazolium dye, apoptosis, cell cycle, and different gene expression analysis.

## 2. Results and Discussion

### 2.1. ABX-CRG_K_ Characterization

#### 2.1.1. Differential Scanning Calorimetry

**Figure 1:**
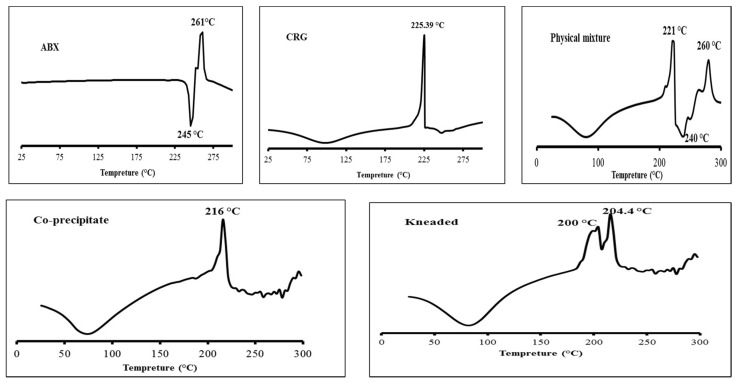
DSC thermo-grams of Heat Flow (mW) on *Y*-axis versus Temperature (°C) on *X*-axis for ABX, CRG_K_, ABX-CRG_K_ as a physical mixture, co-precipitate, and kneaded product.

Differential scanning calorimetry (DSC) thermograms of the different samples are shown in Figure 1. ABX exhibited sharp endothermic and exothermic peaks at 245 and 261 °C, respectively, because of melting and thermal degradation, respectively [15]. CRG_K_ showed a broad endothermic peak between 50 and 150 °C due to loss of free moisture and/or conformational polymer changes. In addition, the polymer underwent thermal degradation, shown as a sharp exothermic transition at 225.39 °C [24]. The ABX-CRG_K_ physical mixture showed a thermal behaviour similar to the single components. However, the drug melting and thermal degradation peaks were slightly downshifted to 237 °C and upshifted to 280 °C, respectively, which could be due to physical interaction between the solid components during heating. The co-precipitate obtained by mixing aqueous solutions of CRG_K_ and ABX to form an ABX-CRG complex preparation yielded a broad endothermic peak in the range of 50 to 150 °C and an exothermic peak at 216 °C; both closely matched with those obtained for CRG_K_ as single components and in the physical mixture. However, the melting and thermal degradation peaks of ABX, also shown for the physical mixture in the range of 225 to 300 °C, were not shown for the co-precipitate. Instead, a fluctuated pattern of endothermic and exothermic small peaks was obtained for the co-precipitate in the same range. This change in the thermal behaviour of ABX suggested that it was molecularly dispersed and amorphised in the co-precipitate, likely due to drug–polymer complexation [17,25]. The corresponding ABX-CRG_K_ kneaded product exhibited similar drug thermal behaviour to the co-precipitate, suggesting drug complexation and amorphisation occurred upon kneading. However, the thermal degradation of CRG_K_ in this product was shown as split, overlapped peaks, which suggested a non-homogeneous product in terms of complexation, probably because while kneading, the polymer was partially dissolved, and ABX-CRG complexation was mainly limited to the gel layer of the polymeric solid particles. In conclusion, the co-precipitate complexation showed a more uniform complexation and crystallization [25]. The co-precipitate complex was further characterized and incorporated into CS and SA mixtures to develop the sustained release nanosuspension formulation.

#### 2.1.2. Powder X-ray Diffraction (PXRD)

**Figure 2:**
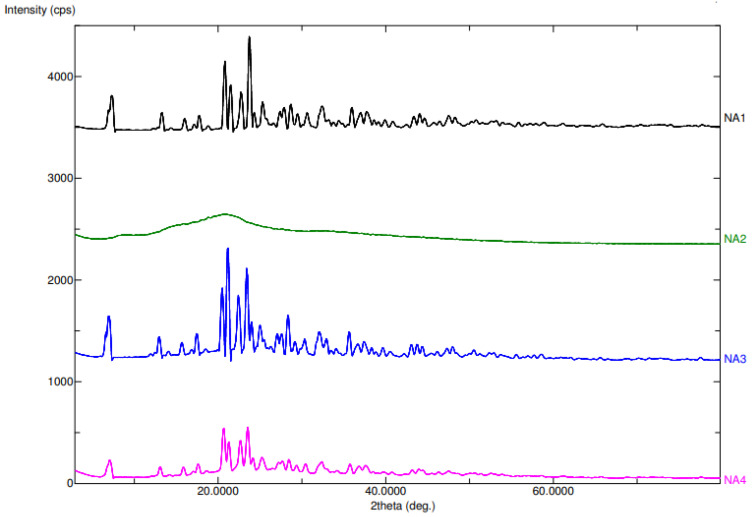
PXRD results of free ABX (NA 1), CRG_K_ (NA 2), physical mixture (ABX + CRG_K_; NA 3), and co-precipitate (NA 4) complex.

The diffractograms for the ABX, CRGK, physical mixture, and ABX-CRGK of the co-precipitated product are shown in Figure 2. For the ABX (NA1), the well-defined peaks indicate the crystalline nature of the free ABX. On the other hand, the CRGK (NA2) diffractogram revealed the amorphous character of this polymer due to the broad but diminished peak of CRGK. The ABX crystalline character was shown in the diffractogram due to presence of sharp peak. However, in the co-precipitated complex preparation, the complex crystalline peaks were similar to the ABX peak in the 2-theta range of 10° to 40°, but the intensity of the complex peaks were half of the free ABX peaks’ intensities. The crystalline nature of the ABX was less than the free ABX and the physical mixture, indicating the amorphization of the ABX using CRGK for the co-precipitate complex preparation [17,25]. This result supported the co-precipitate results in the DSC characterization [17,25].

#### 2.1.3. Scanning Electron Microscopy (SEM)

**Figure 3:**
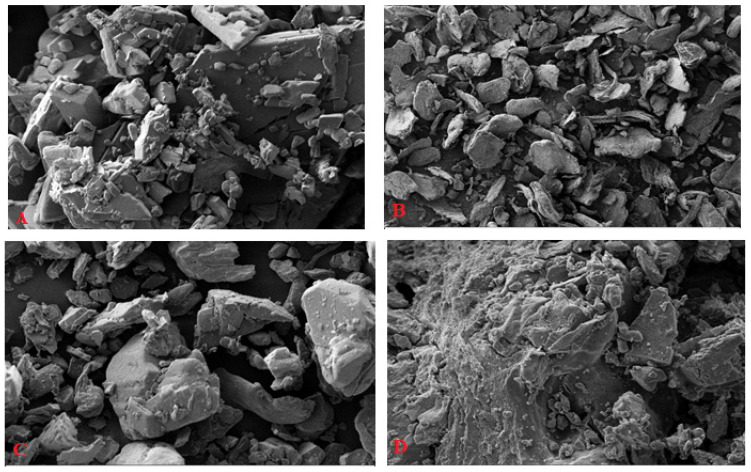
SEM results of ABX (**A**), CRG_K_ (**B**), physical mixture (**C**), and co-precipitate complex (**D**).

The morphology of ABX, CRG_K_, physical mixture, and ABX-CRG_K_ co-precipitate are observed via scanning electron microscopy (SEM) images. The ABX rectangular shape uniform powder is shown in Figure 3A. On the other hand, the non-uniform CRG_K_ polymer powder is shown in Figure 3B. As expected, the physical mixture showed both ABX’s uniform particles and CRG’s non-uniform particle shape (Figure 3C). Furthermore, the ABX-CRG_K_ co-precipitate complex showed more dispersed ABX or CRG_K_, rather than other particles, due to complexation as shown in Figure 3D [25].

### 2.2. Nanosuspension Formulation

At a concentration of 5 mg/mL, ABX was added to the suspensions as a free drug or as an ABX-CRG co-precipitate complex. Four formulations (F1–F4) were prepared using SA as a gel-forming agent to optimize the nanosuspension compositions as shown in Table 1. NA and CRG_K_ were added to slow the ABX-CRGK complex dissociation, and CS served as a cross-linking agent. Different formulations were selected to check the impact of CS removal (F1), CRG_K_ removal (F2), SA solid particles usage instead of its solution (F3), and the impact of combining the CRG_K_, CS, and SA solution to form F4 [26,27]. Furthermore, the selection and optimization of the SA, CS, and CRGK grades and amounts were based on having the ABX particles released as nanoparticles from the polymeric raft/floating gel [25,27].

### 2.3. Drug-Release Study and Gel Floatation

The profiles had an immediate initial release phase in 0.5 h, which was due to ABX free-ABX release followed by a sustained release phase during the interval of 0.5 to 8 h (Table 2).

The optimisation was based on dissolution parameters, which were based on the initial burst release and later sustained release. The burst drug release was evaluated by calculating the initial dissolution rate (IDR), which is defined as the dissolved drug volume (mg) per min for the first 30 min. Additionally, the equation shown below was used to measure the initial mean dissolution times for this duration (MDT_I_) and the SR process from 0.5 to 8 h (MDT_SR_).

$$MDT=\frac{\sum_{i=1}^{n}t_{mid}\cdot \Delta A}{\sum_{i=1}^{n}\Delta A}$$

in which, *i* = the number of dissolution samples, *n* = the number of dissolution periods, *t* = the time at the midpoint between times *t_i_* and *t_i_*_−1_, and Δ*A* = the amount of drug dissolved between times *t_i_* and *t_i_*_−1_. Thus, the time needed for 50% drug release is the MDT. A slower release rate could be reflected by a higher mean dissolution time value [25]. Table 1 list the different formulations and Table 2 shows the drug-release parameters from the previous calculations for the respective suspension formulations. In addition, the goal was to have the least IDR value, highest MDT values (immediate and SR), and gel formation contact with the simulated gastric fluid (0.1 N HCl). As a result, a comparison between the F1 (without CS) and F4 (with CS) was performed to check the CS cross-linking impact of enhancing the SA floating gel formed in the 0.1 N HCl. The F1 had a faster burst release phase than F4 as demonstrated by the IDR and MDT_I_ values (Table 2). The IDR value of F1 was more than the F4 by 1.328 ± 0.0031 mg/min and less than the MDT_I_ of F4 by 0.055 ± 0.0053 h (*p* = 0.002). On the other hand, the SR phase (MDT_SR_) of F4 was more than the F1 by 1.310 ± 0.0021 h (*p* = 0.0013), indicating the importance of CS in the SA insoluble and floating gel as an SR matrix for the complexed ABX. A comparison between the F2 (without CRG_K_ complexation) and F4 (with CRG_K_ complexation) was done to check the influence that CRG_K_ complexation had on enhancing the SR of ABX from the SA floating gel formation. F2 had a faster burst release phase than F4, a finding that was supported by IDR and MDT_I_ (Table 2). The IDR value of F2 was more than the F4 by 0.663 ± 0.0043 mg/min and less than the MDT_I_ of F4 by 0.028 ± 0.0033 h (*p* = 0.003). On the other hand, the SR phase MDT_SR_ of F4 was more than the F2 by 0.192 ± 0.0011 h (*p* = 0.011), which indicated the necessity of ABX complexation for incorporation into the SA insoluble and floating gel as an SR matrix. A comparison between the F3 (SA powder used) and F4 (SA liquid solution used) was done to check the importance of the liquid formulation of SA in enhancing the SR of ABX and the SA floating gel formation in contact with 0.1 N HCl. F3 had a faster burst release phase than F4, a finding that was supported by IDR and MDT_I_ (Table 2). The IDR value of F3 was more than the F4 by 0.675 ± 0.0033 mg/min and less than the MDT_I_ of F4 by 0.043 ± 0.0023 h (*p* = 0.0021). On the other hand, the SR phase MDT_SR_ of F4 was more than the F3 by 0.181 ± 0.006 h (*p* = 0.0071), a finding that indicated the importance of the liquid SA solution and onset of faster gel formation in contact with 0.1 N HCl, which was one of the essential factors in the development of the gastro-retentive drug delivery system.

**Figure 4:**
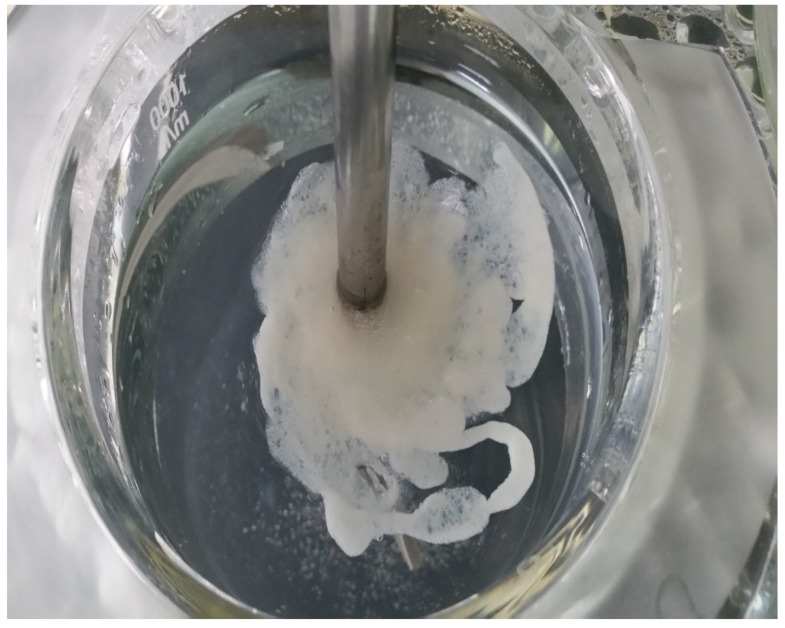
The floating F4 nanosuspension gel after 8 h in 0.1 N HCL (ABX dose of 5 mg/mL).

Interestingly, the F3 and F2 were not statistically significant (*p* > 0.05) with respect to their parameters (Table 2) and similar in their release behaviours, findings that indicated a similar impact of removing CRG_K_ complexation and the use of SA powder. Furthermore, gel formation and floatation of the optimum F4 suspension were optically imaged at 8 h of dissolution. F4 showed rapid gel formation and floatation and was coherent and stable throughout the dissolution study of 8 h (Figure 4). Finally, F4 was aimed to be given orally, which might develop a gastro-retentive floating gel. Additionally, this floating gel would sustain ABX release to act or be absorbed from the formulation [15,17,28]. Afterwards, the rest of the gel might travel to the intestinal area for release and absorption of the remaining ABX. The pH profile for the related formulation was studied [29]. Finally, the critical parameters in the optimum selection were chosen for encapsulating the ABX-CRG complex in alginate with chitosan particles. This formulation had the lowest IDR, highest MDT, and coherent raft/floating gel after contact with the simulated gastric fluid (0.1 N HCl) was selected as an optimized formulation for further characterization and biological evaluation studies. The optimized F4 formulation showed a 95.19% ± 0.54% encapsulation efficiency.

### 2.4. Aging Effect on the Drug Release

The two- and four-week ageing effects on F4 suspension with respect to ABX release specified in dissolution parameters is listed in Table 3. As a result, after two weeks of ageing, the F4 suspension showed an apparent increase in the release rate of the immediate release phase and SR phase with respective *p*-values for the differences of IDR (0.0011), initial MDT (0.0033) and SR MDT (0.0041), which was compared to the freshly prepared F4. On the other hand, four weeks of ageing did not significantly change the parameters of the two weeks aged suspension (*p* > 0.05). The F4 suspension showed a near doubling effect doubling effect on the initial ABX burst release at 0.5 h of dissolution after two weeks of ageing (from 36% to 50%). The drug release was stable between two and four weeks, which might be due to the equilibrium between the free ABX and ABX in complex, which might have been converted to free ABX free in the presence of water. This result supported the previous literature’s data [17,25].

### 2.5. ABX Nanosuspension Characterization

#### 2.5.1. Particle Size Analysis (PSA)

**Figure 5:**
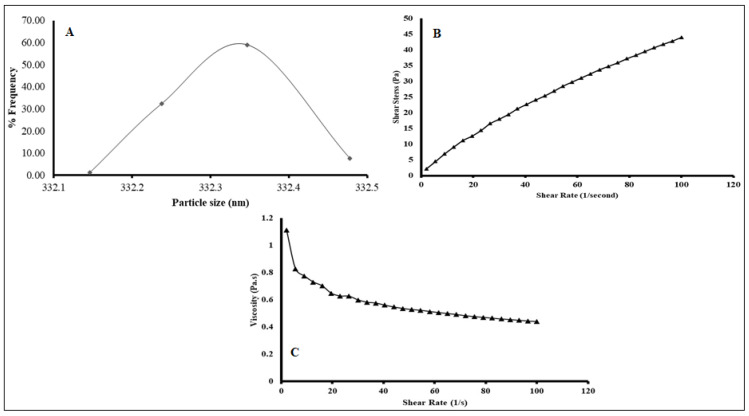
(**A**) Particle size analysis of F4. (**B**) Flow curve of F4 nanosuspension. (**C**) Viscosity-shear rate of F4 nanosuspension.

Freshly prepared F4 or ABX-NS particle size distributions and data summaries are given in Figure 5A and Table 4. The suspension was easily dispersed and had particle sizes ranging from 332.15 to 332.5 nm. The ABX-CRG complex and other insoluble ingredients, such as CS, were subject to high shear homogenization, after which they were dispersed in SA solution with stirring. It seems that homogenization provided enough energy and sheer force for micron grinding of the dispersed particles. SA, as a dispersing solution, can stabilize these fine particles in the final suspensions. Being a hydrocolloid, it can be adsorbed on the surface of the dispersed ground particles in multi-molecular or multi-sheet patterns, which would stabilize the ground particles via steric hindrance [29]. The ABX polymeric nanoparticles resulted from the shearing forces applied from the liquid and high molecular weight SA, CS, and CRGK complexations and incorporation [25,27].

Furthermore, the SA carboxylate groups could charge the adsorbed layers of SA around the dispersed particle; thus, further stabilization can be achieved by electrostatic repulsion. Accordingly, the particle size nature of the suspension was very likely a result of efficient grinding and stabilization of the ground particles by SA that acted as a stabiliser by electro-steric mechanism and minimized flocculation of the particles in the suspensions. Based on the exact proposed stabilization mechanism, SA would also offer physical stabilization of the suspensions upon ageing. They had significant differences in their mean particle size and differed concerning the width and normality of their particle size distributions and polydispersity index (PDI). Size distributions reflect the sizes of the ground ABX-CRG complex if it is assumed that no interactions between the solid components during grinding has occurred. The zeta potential value of the suspension (Table 4) was negative (−42 ± 1.48 mV). The sign of these values could result from carboxylate groups of SA and sulphate groups of CRG. The suggested reason might be that SA and CRG arrangements are on the surface. The zeta potential, representing the surface potential of the electrostatic double layer around dispersed particles, is a measure of repulsion among dispersed particles of equal charge. Absolute potential values higher than 30 mV are required to prevent the flocculation of dispersed particles and achieve a stable dispersion. Values around zero result in rapid flocculation and poor mono-dispersibility of the particles or poor uniform distribution [30,31]. PDI indicates the agglomeration probability. Values < 1 indicate a low agglomeration tendency. As a result, the F4 suspension was easily dispersed upon shaking. SA, as a dispersant, likely contributed to the easy dispersibility by forming a thick electrostatic layer with optimal conformation around the particles.

#### 2.5.2. Viscosity Measurement

According to Figure 5B, the viscosity of the suspension changed significantly as the shear rate increased, and viscosity decreased as a function of the increase in shear stress. Accordingly, the suspension as a liquid followed a non-Newtonian or pseudo-plastic behaviour, which was also confirmed when shear stress was plotted versus shear rate (Figure 5B), and the plot was non-linear with negative deviation. In Figure 5C, the viscosity decrease was exponential, and the highest reduction in viscosity was at a low shear rate of 2 to 5 1/s, after which shear-thinning progressively decreased. Shear thinning happens under several conditions. If the particles in a system aggregate, increasing the shear rate will cause the aggregates to break down leading to a reduction in the immobilized solvent. However, shear thinning could result from the SA being used as a hydrocolloid in the suspension for gel formation. The early discussion of particle size and zeta potential suggesting a high mono-dispersity of the suspensions supported the later reason as the most probable factor for the shear thinning.

When it occurs considerably at a low shear rate, shear thinning is essential for suspension pourability after shaking as mild agitation seems to be enough to induce significant fluidity and pourability. If viscosity upon shaking is not instantly recovered after the shear force is removed, but rather slowly decreased over a certain period, a favourable thixotropic system would be achieved in the suspensions. Thixotropic suspensions have the advantage of being fluid enough for an easy pourability upon shaking and highly viscous upon standing for good stabilization of the dispersed particles against flocculation and sedimentation. The viscosity behaviour of the suspensions could be attributed to the SA that was used as a gel-forming and dispersing agent. As a result, the disentanglement–entanglement processes, the alignment of the SA polymer chain, and the direction of shear were correlated to the thixotropic behaviour of the SA solution. The viscosity behaviour of the SA as aqueous solutions at different concentrations (1–3%) has been previously studied. The solutions that exhibited shear-thinning or pseudo-elastic behaviour following the behaviour of the studied suspension contained 3% SA. In addition, the solutions demonstrated thixotropic behaviour, which was more substantial as the concentration of SA increased [32]. Accordingly, our suspension was thixotropic and thus achieved good pourability upon shaking and good physical stability during shelf-life.

### 2.6. In Vitro Cell Line Study in A549 Cells

#### 2.6.1. Cell Viability Using MTT Assay

**Figure 6:**
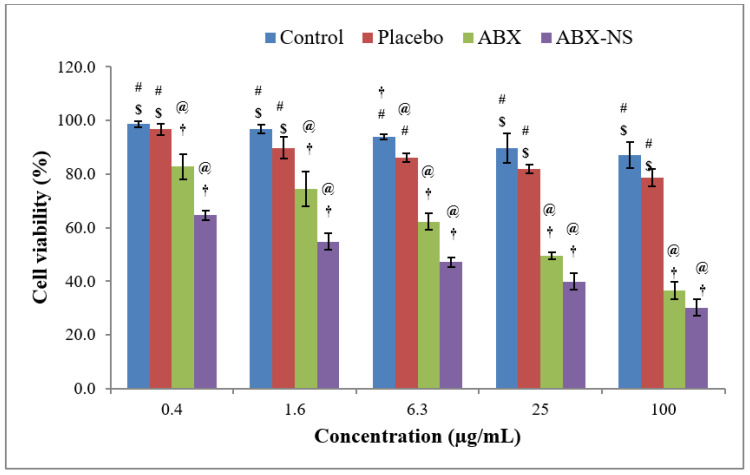
Bar diagram of results of cell viability studies by 3-(4,5-dimethylthiazol-2-yl)-2,5-diphenyl tetrazolium bromide (MTT) assay in A549 cells after treatment with control, placebo, ambroxol (ABX), and ambroxol nanosuspension (ABX-NS) samples. [Statistical inferences: @, *p* < 0.05, compared with control; †, *p* < 0.05, compared with placebo; #, *p* < 0.05, compared with ABX; $, *p* < 0.05, compared with ABX-NS].

A significant decrease in cell viability was shown by the ABX and ABX-NS (F4) samples at all selected concentrations compared to the control and placebo treatments (Figure 6). Thus, both ABX and ABX-NS samples have a significantly higher (*p* < 0.05) cytotoxic effect on the cells. Interestingly, it was further noted that the cell viability in the presence of ABX-NS was significantly lower (*p* < 0.05) than cells exposed to ABX, except at the highest selected concentration of 100 µg/mL (*p* > 0.05). Furthermore, the IC_50_ values were 3.74 and 0.69 µg/mL for ABX and ABX-NS samples, respectively. This type of significant reduction in the cell viability by nanosuspension of a drug compared to the pure drug solution has been previously demonstrated. The enhanced cellular uptake of nanosized particles of the nanosuspensions might be responsible for a significant decrease in cell viability. Meanwhile, slight enhancement of cytotoxicity induced by the pure drug at high concentration has been shown with the use of docetaxel [33]. Such an effect might have also been produced after exposure to the highest concentration of ABX at which the difference in cell viabilities produced by ABX and ABX-NS was less than lower concentrations.

#### 2.6.2. Apoptotic Activity by Flow Cytometry

**Figure 7:**
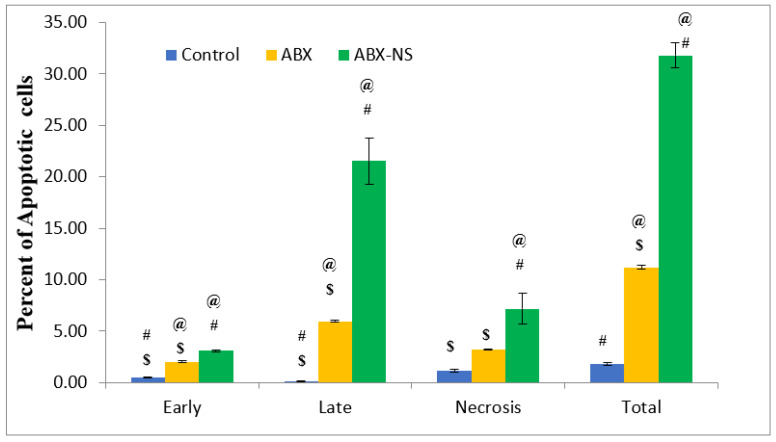
Bar diagram of percent of apoptotic cells in early, late, necrosis, and total stages of apoptosis after treatment with IC50 concentration of control, ambroxol (ABX), and ambroxol nanosuspension (ABX-NS). [Statistical inferences: @, *p* < 0.05, compared with control; #, *p* < 0.05, compared with ABX; $, *p* < 0.05, compared with ABX-NS].

The results from the apoptotic activity showed an increase (*p* < 0.05) in the percentage of cells in the late phase following treatment with ABX-NS (Figure 7). Meanwhile, a significant effect (*p* < 0.05) of ABX in the early phase was detected, but no significant effect (*p*-value > 0.05) in the necrosis phase compared to the control treatment was noted. A significant increase (*p* < 0.05) in the percentages of cells in the early and necrosis phases was also observed with ABX-NS. As a result, the total percentage of cells was highest for the ABX-NS treated cells. A similar higher effect of the nanosized drug delivery system on the late apoptotic phase was observed in a previous study [34].

#### 2.6.3. Cell Cycle Analysis by Flow Cytometry

**Figure 8:**
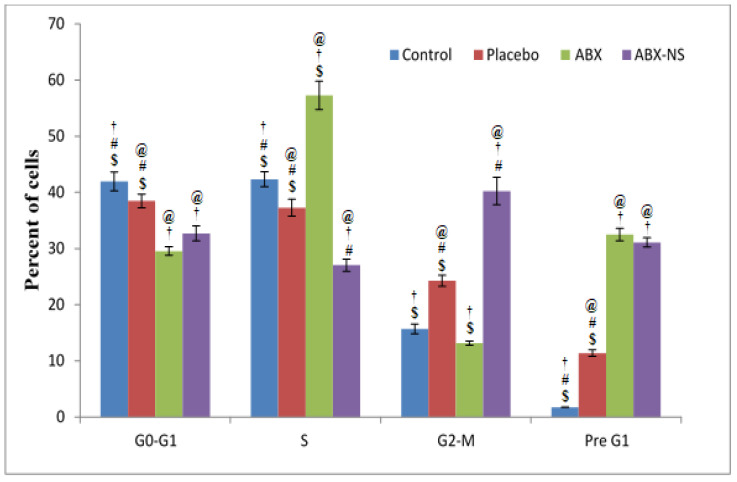
Results of cell cycle analysis, presented as histograms, after treatment with IC50 concentration of control, placebo, ambroxol (ABX), and ambroxol nanosuspension (ABX-NS) treatments in A549 cells. [Statistical inferences: @, *p* < 0.05, compared with control; †, *p* < 0.05, compared with placebo; #, *p* < 0.05, compared with ABX; $, *p* < 0.05, compared with ABX-NS].

The results of the study (Figure 8) show that the ABX-NS sample produced the highest percentage of cells in the G2-M phase. This effect in the G2-M phase was significantly higher (*p* < 0.05) compared to all other samples, including the pure ABX. An increase in the cells in the G2-M phase is a strong indicator of the apoptotic effect. Therefore, the significant apoptotic effect in the ABX-NS sample was confirmed. Meanwhile, it was noted that the ABX increased significantly (*p* < 0.05) when the percentage of cells in the S phase was highest. Cell cycle arrest in the S phase is an important step in apoptosis induction. However, studies with resveratrol have proven that such cell cycle arrest in the S phase cannot always induce apoptosis [35]. Therefore, it may be inferred that ABX is capable of inducing cell cycle arrest in the S phase, but cannot induce apoptosis at a level as significant as that produced by the ABX-NS, a result that might be correlated with the ABX nanoparticles, CRG, SA and CS.

#### 2.6.4. Mitochondrial Membrane Potential Activity (MMP)

**Figure 9:**
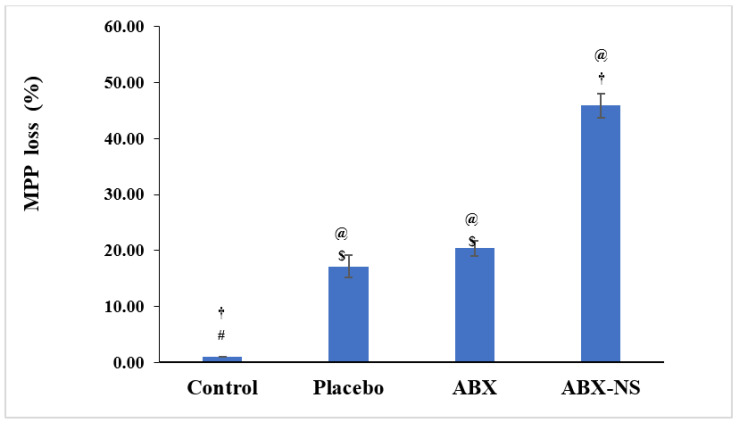
Histograms showing effect of IC50 concentration of ABX, and ambroxol nanosuspension (ABX-NS) treatment on percentage loss of MMP in A549 cells. [Statistical inferences: @, *p* < 0.05, compared with control; †, *p* < 0.05, compared with placebo; #, *p* < 0.05, compared with ABX; $, *p* < 0.05, compared with ABX-NS].

A significant reduction in MMP (*p* < 0.05) in the ABX-NS sample, which amounted to a loss of 45.90% ± 2.15% compared to free ABX 20.3% ± 1.30% (Figure 9). Similar results are reported for 2-methyl-estradiol-loaded polymeric nanoparticles [34]. Thus, ABX-NS can be assumed to affect the integrity of the mitochondrial membrane. The enhanced loss of MMP by ABX-NS compared to ABX showed that the nanosuspension formulation was better, causing an MMP loss, which might have also resulted from the higher apoptotic effect of ABX-NS compared to ABX. Meanwhile, no significant difference in the effects of placebo and ABX (*p* > 0.05) was found. The presence of CRG_K_, CS, and SA in the placebo formulation might have resulted in the MMP loss comparable to free ABX treatment.

#### 2.6.5. Effect of ABX-NS on Caspase-3, NF-kB, IL-6, IL-1β, and TNF-α Activities by ELISA Method

**Figure 10:**
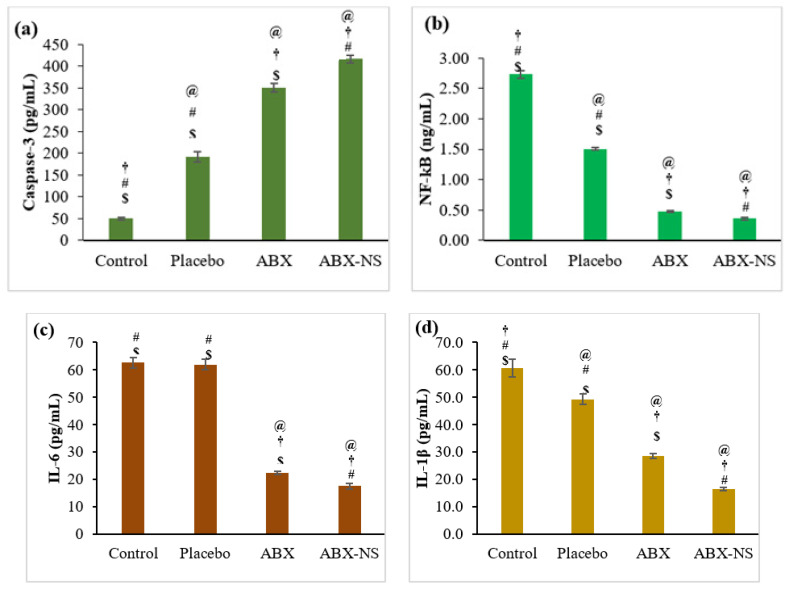

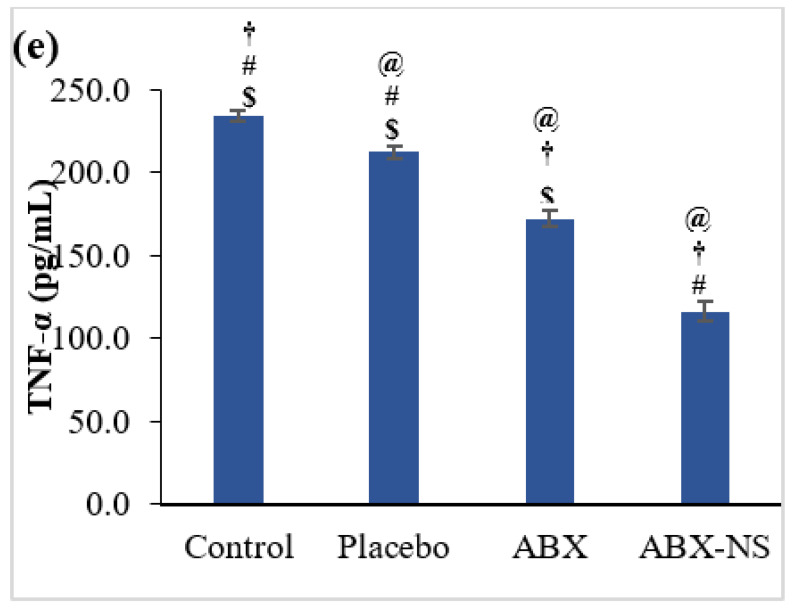
Effect of IC50 concentration of control, placebo, ambroxol (ABX) and ambroxol nanosuspension (ABX-NS) on (**a**) Caspase-3 (**b**) nuclear factor kappa B (NF-kB) (**c**) interleukin-6 (IL-6) (**d**) interleukin-1β (IL-1β) (**e**) tumour necrosis factor—alpha (TNF-α). [Statistical inferences: @, *p* < 0.05, compared with control; †, *p* < 0.05, compared with placebo; #, *p* < 0.05, compared with ABX; $, *p* < 0.05, compared with ABX-NS].

The estimation of biomarkers was carried out to study the effect of ABX-NS on them. An increase or decrease in the concentration on these biomarkers can be used to monitor cytotoxic or apoptotic activity. In the case of caspase-3, its activation, and thereby increased concentration, indicates apoptotic activity. ABX-NS showed a significantly higher caspase-3 concentration than all other treatments (Figure 10a). The caspase-3 concentrations were 416.6 ± 9.11 and 350.8 ± 9.54 pg/mL for ABX-NS and ABS, respectively. Meanwhile, ABX-NS and ABS showed a significant reduction in the NF-kB level. NF-kB is involved in the regulation of IL-1β transcription, which subsequently promotes tumorigenesis. Thus, lower concentrations of NF-kB are indicative of tumour suppression.

Thus, it may be inferred that a significant decrease in NF-kB levels after the treatment with ABX-NS (Figure 10b) is indicative of anti-tumour activity. Furthermore, a significant difference in NF-kB levels after ABX-NS and ABX treatments (*p*-value < 0.05) confirmed that the nanosuspension induces higher anti-tumour activity than the pure drug. IL-6 levels followed a pattern similar to that shown by NF-kB levels. ABX-NS treatment caused a significant decrease in (*p* < 0.05) IL-6 levels compared to the other sample treatments (Figure 10c). Higher IL-6 production can suppress natural killer (NK) cells. However, this process adversely affects intrinsic tumour detection and killing activity of NK cells [36,37]. Hence, a reduction in the IL-6 level induced by ABX-NS can be advantageous by avoiding suppression of NK cells and thereby favouring tumour recognition and killing activity. Furthermore, it was observed that this suppression of IL-6 by ABX-NS was significantly higher than that by ABX (*p* < 0.05). The reduction in IL-6 levels caused by ABX has also been demonstrated in previous studies [38]. Therefore, the formulation of ABX nanosuspension appears to enhance its anti-tumour activity.

Since NF-kB regulates IL-1β transcription, a decrease in IL-1β levels can be expected after ABX-NS treatment due to the reduction in NF-kB levels, a result that was proven to be valid based on the results of the estimation of IL-1β levels (Figure 10d). The IL-1β level was significantly less for the ABX-NS treatment (*p* < 0.05). The concentrations of IL-1β were 16.5 ± 0.5 and 28.6 ± 0.9 pg/mL for ABX-NS and ABX, respectively. Meanwhile, the TNF-α level was lowest after the ABX-NS treatment (Figure 10e). Furthermore, the TNF-α levels of ABX-NS (116.2 ± 6.2 pg/mL) and ABX (172.2 ± 5.2 pg/mL) were significantly different. Similar to IL-6, a reduction in TNF-α levels have also been reported for ABX [38]. Thus, the nanosuspension formulation led to a significant decrease in TNF-α production compared to pure ABX (*p* < 0.05). In general, TNF-α can induce apoptosis and produce cytotoxicity. Thus, the decrease in TNF-α did not appear to be related to the anti-tumour activity of ABX. Nevertheless, TNF-α is considered a double-dealer, as it can also induce cancer cell proliferation and growth [7]. Furthermore, it was observed that the reduction in TNF-α by ABX can provide a protective effect on the lungs [39]. Therefore, a decrease in TNF-α levels induced by ABX-NS may be advantageous in cancer treatment, particularly against LC.

It was noted that the placebo treatment had a significant influence (*p* < 0.05) on the studied biomarkers (except Il-6) compared to control treatment. It has been established that CRG_K_, a major constituent of the placebo formulation, is an inflammatory agent and induces changes in the levels of these biomarkers. Furthermore, CS and SA, the other components present in the placebo formulation, have also been reported to affect these biomarkers. Therefore, the effect of the placebo formulation can be explained. Nevertheless, a significant difference (*p* < 0.05) between the ABX-NS and placebo with respect to their effects on all measured biomarkers.

#### 2.6.6. Effect of ABX-NS on Molecular Markers (*Bax*, *Bcl-2*, and *p53*) Using RT-PCR

**Figure 11:**
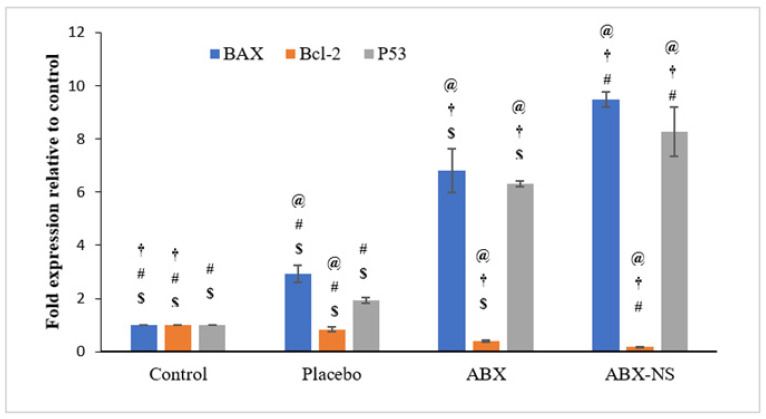
*Bax, Bcl-2,* and *P53* gene expressions after treatment with IC50 concentration of ambroxol (ABX), ambroxol nanosuspension (ABX-NS), control and placebo. [Statistical inferences: @, *p* < 0.05, compared with control; †, *p* < 0.05, compared with placebo; #, *p* < 0.05, compared with ABX; $, *p* < 0.05, compared with ABX-NS].

The results of the expression of the *Bax*, *Bcl-2* and *p53* genes were reported as relevant fold-change (increase or decrease) when compared to control (Figure 11). Treatment with ABX-NS caused a significant increase (9.5 ± 0.29-fold) in *Bax* gene expression in A549 cells (*p* < 0.05). Higher expression of the *Bax* gene was shown to trigger apoptosis [40]. Meanwhile, treatment with ABX caused an increase in the expression of the *Bax* gene by 6.8 ± 0.83-fold in the A549 cells (Figure 11).

Exposure to ABX-NS caused a significant reduction (0.18 ± 0.02-fold; *p* < 0.05) of the *Bcl-2* gene in the A549 cells compared to other treatments. A similar reduction (0.39 ± 0.03-fold level, corresponding to a 0.61 ± 0.03-fold decrease) was observed when these cells were treated with ABX as compared to the control. Thus, the ABX-NS nanosuspension formulation showed higher and significantly inhibitory effects (*p* < 0.05) on the expression of the *Bcl-2* gene in the A549 cells (*p* < 0.05) compared to ABX. Reduced expression in the *Bcl-2* gene has been reported to cause a decrease in apoptosis in both SCLC and NSCLC [41,42]. Thus, a decrease in *Bcl-2* level can favour apoptosis.

The expression of the *Bax and Bcl-2* were also affected (*p* < 0.05) in A549 cells treated with placebo when compared to untreated (control) cells. This result might have been due to the presence of CRG_K_, CS, and SA in the placebo formulation. In this situation, a significant difference (*p* < 0.05) between the ABX-NS and placebo in their effects was detected.

Treatment with ABX-NS caused a higher increase (8.3 ± 0.93 fold; *p* < 0.05) in the expression of the *p53* gene in the A549 cells compared to cell treated with ABX (6.3 ± 0.10-fold increase). Activation of apoptosis can be mediated by *p53* and, therefore, an increase in the expression of *p53* gene indicates a higher probability of triggering apoptosis [43,44].

## 3. Conclusions

The ABX-CRG_K_ co-precipitate complex led to modifications in the crystalline nature and character of ABX. SEM results confirm the dispersion of ABX and CRG_K_ particles in the complex. The drug release study of ABX-NS (F4) and gel flotation confirmed the most optimum suspension with SR character in the simulated gastric media; fast and coherent floating gel formed over 8 h. The biological studies proved that the ABX released from ABX-NS showed substantial cytotoxicity, apoptosis, anti-inflammatory and anticancer activity as compared to free ABX and blank nanosuspension. Thus, the developed sustained release gastro-retentive gel-forming nanosuspension of ABX prepared by co-precipitation was found to be promising for further clinical evaluation.

## 4. Materials and Methods

Ambroxol (ABX), kappa-carrageenan (CRG_K_), sodium alginate (SA), and chitosan (CS) were purchased from Sigma-Aldrich (St. Louis, MO, USA). Adenocarcinomic human alveolar basal epithelial cell (A549) lung cancer cells were purchased from ATCC (Manassas, VA, USA). The A549 human lung cancer cells were cultured in DMEM (Giboco, UK) supplied with foetal bovine serum and penicillin/streptomycin. The cell line was grown at 37 °C under a humidified atmosphere with 5% CO2 to 80% to 90% confluence. The Thiazolyl Blue Tetrazolium Bromide (MTT) reagent kit was purchased from ABCAM, Cambridge, UK. Other chemicals used were of analytical grade. The Annexin V-FITC Apoptosis Detection Kit and Cell cycle kits were purchased from BD Pharmingen (San Diego, CA, USA). Caspase-3, nuclear factor kappa-B (NF-kB), interleukin-6 (IL-6), interleukin-1β (IL-1β), and tumour necrosis factor-alpha (TNF-α) ELISA kits were procured from Invitrogen^®^, Thermo Fisher Scientific, Waltham, CA, USA.

### 4.1. ABX-CRG_K_ Complex Formation

The complexation methods were based on the maximum complexation potential as previously described [17].

#### 4.1.1. Co-Precipitation

Mixing and stirring of the ABX and CRG_K_ (5 mL, 20 mg/mL and 5 mL, 10 mg/mL, respectively) aqueous solutions were performed until a precipitate and cloudy mixture were produced. Afterwards, centrifugation and drying for the precipitate were done. The dry product was crushed using a mortar and pestle to reduce its particle size and recovered with sieving, through a 250 µm sieve [17,25].

#### 4.1.2. Kneading

Equal quantities of the ABX and CRG_K_ powder were mixed in distilled water to obtain a homogeneous paste. Afterward, the paste was treated in the same manner as the co-precipitate [17,25].

### 4.2. ABX-CRG_K_ Characterization

Characterizations of the ABX-CRG_K_ complexes (co-precipitate and kneaded products) were compared to the ABX, CRG_K_, and the materials physical mixtures.

#### 4.2.1. Differential Scanning Calorimetry (DSC)

Powder samples (5–10 mg) were weighed and scanned in sealed aluminium pans with drilled covers at 25 to 300 °C. The instrument was tuned using indium as a standard. A DSC equipment (Mettler-Toledo/Greifensee, Switzerland) was used.

#### 4.2.2. Powder X-ray Diffraction (PXRD)

The samples were analysed using an X-ray diffractometer (Maxima XRD-7000X, Shimadzu, Kyoto, Japan) equipped with a Cu anode and a 2.2 kW tube. A goniometer in theta-2 theta geometry with a Goebel mirror was used for parallel beam radiation and Cu Kb reduction.

#### 4.2.3. Scanning Electron Microscopy (SEM)

SEM (FEI Inspect F50, FEI, Tokyo, Japan), was used to investigate the morphology and distribution of the powders. The dried specimens were screened at an accelerating 30 kV voltage when placed on a metal stub (with double-sided adhesive tape).

### 4.3. Nanosuspension Formulation

At a 5 mg/mL concentration, ABX was added to the suspensions as a free drug or as an ABX-CRG co-precipitate complex. Four formulations (F1-F4) were prepared using SA as a gel-forming agent to optimize the suspension compositions, as shown in Table 1. SA and CRG_K_ were to slow the ABX-CRG_K_ complex dissociation, and CS served as a cross-linking agent. Table 1 shows the coding information for the formulations. Aqueous SA solutions with high molecular weights (3%) were prepared for F1, F2, and F4. In a glass beaker containing 8 mL distilled water, ABX (100 mg) or ABX-CRG_K_ co-precipitate complex (200 mg) and CS (10 mg/mL) was placed and homogenized using a homogenizer. The homogenized mixtures (8 mL) were mixed and stirred with the 12 mL of 3% SA solution [14]. For F3, the dry components in Table 1 were mixed to have a dry physical mixture.

### 4.4. In Vitro Drug Release Study

At 50 rpm and 37 ± 0.5 °C, a Type II (paddle) dissolution apparatus (Erweka, Langen (Hessen), Germany) was used, and 0.1 N HCl was the dissolution medium (900 mL). Suspension volumes (15 mL) or dry weight equal to 75 mg of ABX were used in the experiments. F1, F2, F3, and F4 formulations were placed using plastic syringes, threadlike inserted into the dissolution medium. Samples of 5 mL during dissolution were taken at fixed time intervals for 8 h and immediately replaced with equal volumes of fresh medium. The UV-absorbance of ABX at 310 nm was measured in the samples. The ABX concentrations were calculated using a calibration curve of ABX in 0.1 N HCl. Additionally, the optimum suspension (F4 or ABX-NS) gel floatation was observed using images at 8 h of dissolution [17,45]. Furthermore, the encapsulation efficiency of the optimum nanosuspension formulation was determined using the UV-Spectroscopy instrument (Shimadzu, Kyoto, Japan). Hence, the optimum suspension was diluted two-fold using distilled water and centrifuged to obtain the supernatant layer [46]. ABX concentrations were measured at 310 nm in triplicate [17]. The four nanosuspension formulations were prepared and optimized formula was selected based of in vitro drug release study. The formulation with the lowest IDR, highest MDT, and coherent raft/floating gel in contact of the simulated gastric fluid (0.1 N HCl) was selected as an optimized formulation for further characterization and biological evaluation studies.

$$Encapsulation\;efficiency=\frac{Total\;drug\;amount-Supernatant\;drug\;amount}{Total\;drug\;amount\;\left(mg\right)}\times 100\%$$

### 4.5. Aging Effect on the Drug Release

The optimized suspension F4 or ABX-NS was maintained at room temperature in amber glass bottles. The ageing test was performed using a dissolution type II apparatus for two weeks and stored for one month.

### 4.6. ABX Nanosuspension Characterization

#### Particle Size Analysis (PSA) and Viscosity Measurement

The Nanoparticle Size Analyser was used to determine the particle size, zeta-potential, and polydispersity index (PDI) of the colloidal systems (NANOTRAC WAVE II/Q/ZETA, Microtrac, Osaka, Japan). The Particle size analysis for F4 assessed the sizes of the ABX released particles from the raft formed in the dissolution study. For the measurement of viscosity, an MCR 301 rotational rheometer (Anton Paar, Graz, Austria) was used. F4 sample volume (3.8 mL) was mounted in a coaxial cylinder (gap width 1.128 mm) at 25 °C 0.01 °C. The shear rate was increased from 2 to 100 s^−1^, and the shear stress and viscosity were calculated in 29 stages. The time of a one-point measurement was 5 s. Three measurement intervals were recorded. The flow curve was produced by software version 4.2 (Rheoplus) as a function of shear stress (Pa) versus shear rate (1/s). Furthermore, the viscosity (Pa.s) versus shear rate (1/s) curve was plotted.

### 4.7. In Vitro Cell Line Study in A549 Cells

#### 4.7.1. Cell Viability Assay

The A549 human lung cancer cells were used in the cell viability assay, which was determined using the MTT cell proliferation kit. The A549 human lung cancer cells were plated in wells of a 96-well plate (5 × 10^3^ cells/well) and incubated at 37 °C for 24 h in a humidified 5% CO_2_ incubator. The authors replaced the F4 formulation code with the ABX-NS formulation in biological testing. Following this protocol, the A549 cells were treated with ABX-NS, ABX, placebo, or control (without any treatment) corresponding to pre-determined ABX concentrations for 24 h at 37 °C (0.4–100 µg/mL). After 24 h, the culture supernatant was removed and replaced with 100 µL dimethylsulphoxide (DMSO) to solubilize the formazan. The plate was incubated at 37 °C overnight in a humidified 5% CO_2_ incubator for 4 h. Following this step, the absorbance at 570 nm was measured using a microplate reader. The results of the cell viability study, done in triplicate, was reported [46].

#### 4.7.2. Apoptotic Activity by Flow Cytometry

The apoptotic activity was studied using a previously described method [47]. Briefly, the A549 cells (1 × 10^5^ cells/well) were incubated with IC_50_ concentration of ABX and ABX-NS, placebo, or control (without any treatment) for 24 h. These cells were recovered by centrifugation, washed with phosphate-buffered saline (PBS), and resuspended in 1X binding buffer (500 μL) (BD Bioscience, Bergen, NJ, USA). Following this step, Annexin V-fluorescein isothiocyanate (FITC) and PI (5 μL each) were added, and the treated cells were incubated at room temperature for 5 min in the dark and analysed using a flow cytometry (FACS Calibur, BD Bioscience, Bergen, NJ, USA). The resulting data were analysed using the Multicycle software vesio 1.3 (Phoenix Flow Systems, San Diego, CA, USA).

#### 4.7.3. Cell Cycle Analysis by Flow Cytometry

The procedure used to perform the apoptotic activity using the flow cytometer was also used for the cell cycle analysis.

#### 4.7.4. Mitochondrial Membrane Potential Activity (MMP)

MMP measurements were obtained using an assay kit with tetramethylrhodamine methyl ester (TMRM) as the probe [46]. Briefly, the A549 cells were plated in wells of a 96-well plate (1.5 × 10^4^ cells/well) and exposed to IC_50_ concentration of ABX, ABX-NS, and placebo, for 24 h in a humidified 5% CO_2_ incubator. After 24 h, the culture medium was replaced with a solution containing TMRM, and the plate was incubated in the dark. Following this step, the probe solution was removed and replaced with a live-cell imaging buffer before analysis using a flow cytometry.

#### 4.7.5. Effect of ABX-NS on Caspase-3, NF-kB, IL-6, IL-1β, and TNF-α Activities as Measured by the ELISA Method

Caspase-3, NF-κB, IL-6, IL-1β, and TNF-α were quantified using commercial enzyme-linked immunosorbent assay (ELISA) kits (Invitrogen^®^, Thermo Fisher Scientific, Waltham, CA, USA). Briefly, A549 cells were plated in wells of a 96-well plate (5 × 10^4^ cells/well) and treated with IC_50_ concentration of ABX, ABX-NS, and placebo. Untreated A549 cells were included as control. Following 24 h of incubation, 100 μL of the culture medium from each well was mixed with the reagent in the ratio 1:1, mixed for 30 s (500 rpm), and stored at ambient temperature for 30 min. Following this step, the amount of caspase-3, NF-κB, IL-6, IL-1β, and TNF-α were quantified using the commercial ELISA kit following the manufacturer’s recommended protocol.

#### 4.7.6. Effect of ABX-NS on Molecular Markers (Bax, Bcl-2, and p53) Using RT-PCR

The expression of the *Bcl-2*, *Bax*, and *p53* genes were determined using the quantitative polymerase chain reaction (qPCR) approach [34]. Briefly, the A549 cells were plated in wells of a 6-well plate (1 × 10^6^ cells/well) and treated with IC_50_ concentration of ABX, ABX-NS, and placebo. Total RNA was extracted from treated and untreated (control) cells using an RNA extraction kit (QIAGEN, Germantown, MD, USA). The RNA was mixed with the green PCR master mix (QIAGEN, Germantown, MD, USA) and analysed using the Rotorgene RT- PCR system (Biorad, Hercules, CA, USA). The resulting data were analysed using the Rotor-Gene software version 1.7 (Biorad, Hercules, CA, USA). Table 5 displays the details of primers used in the study. The glyceraldehyde 3-phosphate dehydrogenase (GAPDH) gene was used as the housekeeping gene for data normalisation.

### 4.8. Statistical Analysis

The experiments (*n* = 3) were carried out, and the results are reported as mean ± standard error (SE) and standard deviation (SD). A one-way analysis of variance (ANOVA) determined the statistical significance followed by Tukey multiple comparison test with a *p* value < 0.05 considered significant.

## Figures and Tables

**Table 1:** Composition of raft-forming aqueous suspensions of ABX or ABX-CRG_K_ co-precipitate complex prepared at ABX concentration 5 mg/mL suspension.

#	SA-Level	CS-Level (mg/mL)	Free ABX or ABX-CRG_K_ Complex	Physical Status
F1	18 mg/mL	-	ABX-CRG_K_ complex	Liquid Suspension
F2	18 mg/mL	10 mg/mL	Free ABX	Liquid Suspension
F3 *	0.42 mg/mg	0.23 mg/mg	ABX-CRG_K_ complex	Dry physical mixture
F4	18 mg/mL	10 mg/mL	ABX-CRG_K_ complex	Liquid Suspension

F3 *: 0.12 mg/mg of ambroxol.

**Table 2:** Parameters of ABX release in 0.1 N HCl from different raft forming suspensions of ABX-CRG complex using type II (paddle) dissolution apparatus.

Formulation	IDR (mg/min)	MDT_I_ (h)	MDT_SR_ (h)
F1	1.668 ± 0.005	0.149 ± 0.002	1.847 ± 0.001
F2	0.983 ± 0.002	0.176 ± 0.004	2.965 ± 0.003
F3	0.995 ± 0.004	0.161 ± 0.003	2.976 ± 0.004
F4	0.320 ± 0.003	0.204 ± 0.001	3.157 ± 0.005

Each value is the average of *n* = 3 ± standard error.

**Table 3:** Drug release parameters of ageing for the suspension.

Formula Number	IDR * (mg/min)			MDT_Initial_ * (h)			MDT_SR_ * (h)		
	0	2 Weeks	4 Weeks	0	2 Weeks	4 Weeks	0	2 Weeks	4 Weeks
4	0.320 ± 0.041	0.686 ± 0.013	0.707 ± 0.013	0.204 ± 0.043	0.144 ± 0.013	0.147 ± 0.013	3.157 ± 0.03	2.481 ± 0.012	2.471 ± 0.012

* Average of *n* = 3 ± standard error.

**Table 4:** Particle size analysis of F4 nanosuspension.

Nano-Suspension	Mean/Average ^1,2^	Median ^2^	Mode ^2^	Minimum ^2^	Maximum ^2^	Range ^2^	Zeta Potential (mV) ^1^	PDI
F4	332.3 ± 0.14	332.5	332	332.14	332.47	0.33	−42 ± 1.48	0.41

^1^ Mean ± standard error. ^2^ The unit of measurements (particle size) is nm. Range = Maximum − Minimum.

**Table 5:** Primers used in the RT-PCR analysis.

Gene		Primer Sequence
*Bax*	F	5′-TGGCAGCTGACATGTTTTCTGAC-3′
	R	5′-TCACCCAACCACCCTGGTCTT-3′
*Bcl-2*	F	5′-TCGCCCTGTGGATGACTGA-3′
	R	5′-CAGAGACAGCCAGGAGAAATCA-3′
*p53*	F	5′-GACGGTGACACGCTTCCCTGGATT-3′
	R	5′-GGGAACAAGAAGTGGAGAATGTCA-3′
*GAPDH*	F	5′-AATGCATCCTGCACCACCAA-3′
	R	5′-GATGCCATATTCATTGTCATA-3′

F: Forward; R: Reverse.

## Data Availability

The data presented in this study are available in article.
